# Peripartum Depressive Symptoms in Fathers during the COVID-19 Pandemic

**DOI:** 10.3390/jcm13061772

**Published:** 2024-03-20

**Authors:** Silvia Cimino, Luca Cerniglia

**Affiliations:** 1 Department of Dynamic, Clinical and Health Psychology, Sapienza, University of Rome, 00185 Rome, Italy; silvia.cimino@uniroma1.it; 2 Faculty of Psychology, International Telematic University Uninettuno, 00186 Rome, Italy

**Keywords:** COVID-19, feeding interactions, depressive symptoms, fathers

## Abstract

**Background**: This research investigates peripartum depression in fathers during COVID-19, focusing on how pandemic-related distress is associated with paternal depressive symptoms and the quality of father–child feeding interactions. The primary objective was to understand if the pandemic has influenced depressive symptoms in new fathers and how these symptoms impact their interactions with their children, especially during feeding. **Methods**: Utilizing a cross-sectional design, the research employs online surveys and remote observation to gather data from 243 Italian fathers. The analysis involves established psychometric tools like the Symptoms Check-List/90R and the Peritraumatic Distress Index to assess the severity of depressive symptoms and their correlation with father–child interaction exchanges, observed through the SVIA. **Results**: The fathers’ peritraumatic distress due to COVID-19 was significantly and positively associated with the level of their depressive symptoms and negatively correlated with the quality of their feeding interactions with their children. Moreover, elevated levels of peritraumatic stress were notably predictive of higher instances of depressive symptoms in the fathers. Further, higher levels of stress associated with COVID-19 were predictive of a poorer quality of father–child interactions. **Conclusions**: This research highlights the need for inclusive perinatal support programs, offering foundational insights into paternal mental health during pandemics.

## 1. Introduction

Individuals who are transitioning to parenthood are often described as living a multifaceted and profound life change. This transition involves significant psychological adjustments as individuals redefine their identities and relationships. Other authors, such as Schadler [1], emphasized the concept of parental role attainment, a process in which new parents develop their new identity. This period is also marked by heightened stress and adjustment challenges, as outlined by Twenge et al. [2], who underscored the impact on marital satisfaction and individual well-being. Importantly, Lee et al. [3] discussed the crucial role of social support in moderating the stress associated with becoming a parent, highlighting the need for a robust support system during this critical life stage.

A large bulk of research has demonstrated that the COVID-19 pandemic has impaired both the societal and familial capacity of supporting individuals in trouble due to practices like social distancing (even among members of the same family), closing of unnecessary activities, and lockdowns, and intensified mental health issues, such as depressive and anxiety symptoms, in the general population [4].

Even in normal conditions, depressive symptoms emerging after delivery are the most frequently encountered states among new mothers [5]. The DSM-5 characterizes them as occurring within four weeks following childbirth, yet some interpretations extend this period to any point during the first year post-delivery [6].

Although the vast majority of research has focused on mothers, depression is known to impact both mothers and fathers during the period following childbirth [7]. While the majority of studies have concentrated on the effects of maternal postnatal depression on child development, evidence from meta-analyses also shows that fathers are affected, with around 5% diagnosed with major depressive disorder. Consolidated research has shown that fathers may manifest depressive symptoms due to a number of mechanisms. They may suffer from low social support, negative economic situations, a poor quality of marital relationships, discrepancies between prenatal expectations and postnatal realities, and reduced sleep quality [8].

Children of fathers experiencing postnatal depression face a higher risk of negative psychosocial development [9]. Various potential mechanisms through which parental depression might influence child development have been identified, with evidence pointing to the critical and alterable nature of parenting skills such as sensitivity and responsiveness [10].

Furthermore, existing studies have established a link between paternal depression and diminished parenting effectiveness [11]. Despite this, only a limited number of studies have focused on the interactions between fathers and infants in the initial months post-birth, a crucial period for the establishment of lasting interaction patterns. Research by Sethna et al. [12] demonstrated that depressed fathers showed less intrusiveness (a form of disengagement) during free-play sessions with their 12-month-old children [13]. In a similar vein, research has shown that fathers suffering from depression, compared to their non-depressed counterparts, are less likely to engage in activities like reading to their one-year-old infants [13] and demonstrate decreased tactile and vocal stimulation with their infants aged between 2.5 and 4 months [14]. These findings suggest that paternal depression might lead to reduced engagement with infants, although some studies have not found a consistent link [15]. It is important to note, however, that many of these studies are constrained by methodological limitations, particularly the reliance on self-reported data for depressive symptoms and parenting behaviors, which could lead to biases in the results.

The outbreak of SARS-CoV-2 (COVID-19) could possibly have altered the dynamics of the paternal presence at home and their involvement in parenting. The extensive lockdowns spanning two years created an unprecedented opportunity for fathers to significantly impact their children’s development [16]. While increased paternal engagement can positively affect infant development, this period also saw fathers grappling with heightened financial, workplace, and childcare pressures [17]. Preliminary studies suggest that these global lockdowns adversely affected parental mental health and well-being. However, the exact nature of how the pandemic has reshaped parenting practices and family life is still unclear [18].

One particular branch of research has focused on the characteristics of parent–child interactions during feeding as associated with adaptive/maladaptive emotional–behavioral outcomes in offspring. A limited, yet existing, literature has considered the quality of mother–child interactions during the pandemic. However, to the best of our knowledge, no research has given attention to the possible negative impact of COVID-19 on fathers, nor to the associated outcomes in their exchanges with their children. It is important to note that Stern [19,20] highlighted the significance of examining the dynamics of play and feeding in understanding the very nature of the parent–infant relationship. On the other hand, some researchers argue that during feeding, the inability of the parent–infant dyad to engage in mutually attuned and sensitive interactions may hinder the infant’s learning of affect self-regulation, potentially leading to maladaptive emotional and behavioral symptoms over time [21].

An increasing body of research indicates the significant influence of positive interactions between fathers and infants on the cognitive and socioemotional growth of children. Such interactions include active engagement, supervision, and the establishment of boundaries [22]. Additionally, fathers indirectly contribute to their child’s development through factors like influencing mother–infant interactions, supporting maternal decisions and behaviors, and providing financial stability [23]. A theoretical perspective highlighting the distinctiveness of father–child interactions compared to mother–child interactions has been posited. This distinctiveness, often characterized by physical play and tactile engagement, plays a crucial role in aiding a child’s development of emotional regulation [24,25].

This research sought to delve into how COVID-19 impacted (a) fathers’ depressive symptoms and (b) the quality of father–child interactions during feeding.

Our hypothesis posited that a higher impact of COVID-19 would correlate with increased levels of paternal depression. Furthermore, it was anticipated that paternal depression would be linked to less favorable interactions with their children. Lastly, it was expected that depressive symptoms in fathers would be indicative of poorer emotional–behavioral functioning in offspring.

## 2. Materials and Methods

### 2.1. Sample

During the second wave of COVID-19 (from 15 November 2020 to 15 March 2021), N = 243 fathers of newborn children were recruited for this study. Recruitment occurred through social media platforms like Facebook and announcements on online psychology research portals. We utilized online consecutive sampling for data collection. Participating parents provided written informed consent, detailing the study’s procedures, following the guidelines of the Ethical Committee of the Department of Dynamic and Clinical Psychology at Sapienza University of Rome (protocol N. 809/2020) and aligned with the Declaration of Helsinki.

Eligibility criteria included having newborn children, being free from physical and mental health issues, and not undergoing psychiatric or psychological treatments (with regards to fathers). After removing 3 fathers with mental/physical disabilities, 1 parent of children with physical conditions, 2 fathers in psychological/psychiatric treatment, and 24 parents who did not complete the assessment, the final sample comprised 213 parents and their children.

### 2.2. Procedure

All procedures took place remotely through online surveys and remote observation.

Fathers were requested to complete an online survey through a digital platform, which encompassed providing their written consent for participation before asking any other questions. This survey included a custom-designed questionnaire to gather sociodemographic data and the impact that COVID-19 had on individuals (Risk Index) (created and used in previous research, see: [26]). Fathers also filled out the Symptoms Check-List/90R (SCL-90/R) [27] to identify possible psychopathological symptoms and the Peritraumatic Distress Index (CPDI) [28] to measure the impact of the pandemic. Moreover, father–child feeding interactions were remotely video-recorded (20 min videos) following a validated method described below (Scala di Valutazione delle Interazioni Alimentari, SVIA; Lucarelli et al. [29]). To avoid fatigue, subjects were instructed to complete all the parts of the digital battery on two different days, with all questionnaires to be filled out on one day and the remotely recorded videos on another day. The order of administration of these measures was randomly selected through a computerized procedure.

### 2.3. Measures

The Peritraumatic Distress Index (CPDI) [28] is a self-reported questionnaire for the evaluation of peritraumatic distress symptoms due to COVID-19. Specifically, items assess symptoms of anxiety, depression, specific phobia, avoidance, and/or compulsive behaviors, in accordance with criterion A for Post-Traumatic Stress Disorder (PTSD). A total score in the range of 0–100 is created by summation. Higher scores are indicative of higher levels of psychological distress. The CPDI showed good internal coherence both in previous studies and in the present one (Cronbach alpha = 0.87).

The SCL-90-R [27] is a widely used 90-item self-reported instrument that assesses psychological symptoms and distress in adults. It employs a Likert scale ranging from 0 (not at all) to 4 (extremely) for responses. This tool is prevalent in both clinical settings and general population studies for the screening and evaluation of psychological symptoms in adults. It encompasses nine scales: Somatization, Obsessive–Compulsivity, Interpersonal Sensitivity, Depression, Anxiety, Hostility, Phobic Anxiety, Paranoid Ideation, and Psychoticism, along with a Global Severity Index (GSI). The Italian version [30] of this measure has demonstrated strong reliability, with Cronbach’s alpha values ranging from 0.70 to 0.96.

The SVIA [29], an Italian version of the original Feeding Scale [31], is applicable to parent–child dyads involving children aged 0 to 36 months. It is designed to assess the quality of parent–child interactions during meal times, both in the case of breastfeeding and of bottle feeding (as in this study). These interactions are typically video-recorded for a minimum duration of 20 min, after which various behaviors and emotional states are systematically coded and analyzed. The SVIA comprises 41 items across four subscales, which include (1) Parent’s Affective States, (2) Interactive Conflict, (3) Food Refusal Behavior, and (4) Dyad’s Affective State. In this study, a unidimensional scale was used, composed of the sum of the four subscales, as suggested by Lucarelli et al. [29]. Higher scores on this scale indicate more pronounced relational challenges. In this study, the tool proved to be reliable, exhibiting good internal consistency, with Cronbach’s alpha values between 0.79 and 0.96.

### 2.4. Analyses Plan

All analyses were performed using SPSS software, Version 26. Preliminary statistical analyses were conducted using descriptive statistics (reliability of the measures, frequencies, mean scores). To compute a COVID-19 Risk Index score from our ad hoc questionnaire, a score (0, 0.5, 1, or 2 points) was assigned to each item based on the degree to which the related change due to COVID-19 affected parents’ psychological–adaptive functioning. The score for each item was summed to obtain a total COVID-19 Risk score. Then, to determine initial significant correlations between study variables, Pearson’s correlation analyses were carried out. Based on the significant correlations that we found, hierarchical multiple regression analyses were carried out to identify the main effects of parents’ peritraumatic distress due to COVID-19, peritraumatic distress, and depressive symptoms and the quality of father–child interactions during feeding.

## 3. Results

The fathers’ and children’s average ages were, respectively, 41.14 years (SD = 7.14) and 5.42 weeks (SD = 3.21), and 51.3% of the children were female. All participants resided in Italy; the majority of the fathers were married (82.1%) and had a high school education (48.2%) or higher (53.2%). Most fathers reported a household income between EUR 55,000 and 75,000 annually.

Our results showed that fathers’ peritraumatic distress due to COVID-19 was significantly and positively associated with the level of their depressive symptoms and positively correlated with the quality of their feeding interactions with their children (Table 1).

### Influences of Parents’ Peritraumatic Distress from COVID-19 on Fathers’ Depressive Symptoms and on the Quality of Feeding Interactions

Utilizing the significant correlations identified earlier, hierarchical multiple regression analyses were performed to determine the predictive nature of the fathers’ peritraumatic distress related to COVID-19, fathers’ depressive symptoms, and on the quality of feeding interactions.

As indicated in Table 2, elevated levels of peritraumatic stress were notably predictive of higher instances of depressive symptoms in fathers. Moreover, higher levels of stress associated with COVID-19 were predictive of a poorer quality of father–child interactions.

## 4. Discussion

The COVID-19 pandemic has significantly impacted mental health worldwide, with a notable increase in depressive symptoms among various populations, including parents. The study by Haydon and Salvatore (2021; [32]) is particularly relevant in this context. Their research in a U.S. community sample that was assessed before and during the initial surge of COVID-19 found that pandemic disruptions were associated with significantly higher depressive symptoms. Their study aligns with our findings. In fact, our results showed that fathers’ peritraumatic distress due to COVID-19 was significantly and positively associated with the level of their depressive symptoms and positively correlated with the quality of their feeding interactions with their children. The pandemic has created unique stressors, such as fear of the virus, economic uncertainties, and disruptions to daily life, which can exacerbate or trigger depressive symptoms. The increased responsibilities at home, including childcare due to school closures and the need to balance work and family life, can be particularly overwhelming for fathers, potentially leading to heightened levels of stress and depression. This is a critical area for further investigation, as paternal mental health significantly impacts family dynamics and child development [33,34,35,36,37].

The negative correlation between peritraumatic distress and the quality of feeding interactions that was found in our study is consistent with existing research emphasizing the importance of parental mental health in parent–child relationships. Sethna et al. (2017; [38]) highlighted the crucial role of father–child interactions in children’s cognitive development. This study extends this understanding by suggesting that peritraumatic distress, particularly during the pandemic, may compromise the quality of paternal interactions during critical activities like feeding. This is significant, because feeding interactions are not just about nutritional intake but also about emotional bonding, communication, and the establishment of trust and security between the parent and child. Depressed fathers may find it challenging to engage in these nurturing behaviors, which can have long-term implications for the child’s emotional and social development. It is important to note that the early years of a child’s life are critical for their overall development, and the quality of parental interactions during this time can have lasting effects.

The broader implications of this study’s findings for family dynamics during the pandemic are significant. The increased stress and mental health challenges that were faced by parents can affect not only their well-being but also their ability to engage effectively with their children. This is particularly relevant in the context of the unique pressures brought about by the COVID-19 pandemic. Studies examining the impact of the pandemic on family life and parenting practices, such as those by Trumello et al. (2021; [39]) and Petts et al. (2021; [40]), have highlighted the reshaping of traditional family roles and responsibilities during the pandemic. The increased home confinement provided fathers with an unprecedented opportunity to be more involved in childcare and domestic responsibilities. However, this increased involvement comes with its own set of challenges, as fathers may also be dealing with job insecurities, financial pressures, and the stress of navigating the pandemic. These factors can strain family relationships and dynamics, potentially leading to increased conflict, reduced marital satisfaction, and altered parenting practices.

The hierarchical multiple regression analyses, which explored the predictive nature of fathers’ peritraumatic distress related to COVID-19 on depressive symptoms and the quality of father–child feeding interactions, align with and extend the existing literature on parental mental health and its impact on parenting and child development. The finding that peritraumatic stress in fathers predicts higher instances of depressive symptoms is consistent with the broader literature on the impact of stress and trauma on mental health. For instance, a study by Allenou et al. (2009; [41]) found that parents who experienced traumatic events, such as motor vehicle accidents involving their children, showed significant levels of peritraumatic distress and depressive symptoms. This suggests that the unique stressors of the COVID-19 pandemic, which can be considered a collective traumatic event, could similarly lead to increased depressive symptoms in fathers.

The finding that higher levels of stress associated with COVID-19 predict a poorer quality of father–child interactions is particularly significant. This is in line with research by Olhaberry et al. (2022; [42]), which highlighted the impact of parents’ psychological difficulties, including depressive symptoms, on the sensitivity of their response toward their children and the quality of triadic interactions. Our study adds to this by specifically linking the unique stressors of the pandemic to a decline in the quality of paternal interactions during feeding, a critical activity for child development.

These findings are situated within a broader context where paternal mental health is increasingly recognized as crucial for family dynamics and child development. Research by Li et al. (2021; [43]) on new fathers in China found that perceived stress, neuroticism, and psychological inflexibility were significant predictors of depressive symptoms. This underscores the complex interplay of psychological factors in the context of fatherhood and highlights the need for targeted interventions to support fathers, especially during unprecedented stressors like a pandemic.

This study has some limitations. First, although using an observational measure to assess the quality of father–child feeding interactions, it relies on self-reported tools for depressive symptoms and parenting behaviors, which could introduce biases. Second, this research focuses exclusively on Italian fathers, which may limit the generalizability of the findings to other cultural contexts. Third, the study uses a cross-sectional design, which limits the ability to infer causal relationships and understand the long-term impact of paternal depression. Fourth, maternal characteristics have not been evaluated, whereas it has been demonstrated that the mental health of mothers significantly affects fathers, shaping parent–infant interactions and the overall family environment [44]. Lastly, the survey was conducted through social networks, and therefore, we are not sure of the correctness and validity of the answers. Notwithstanding these limitations, the study has a number of strengths. It adopts an innovative focus by addressing a less-explored aspect of postpartum depression, concentrating on fathers. Moreover, it is timely and relevant, as it investigates the impact of a significant global event, COVID-19, providing contemporary relevance. Finally, it utilizes established psychometric tools and a comprehensive statistical approach, ensuring data reliability and validity.

## 5. Conclusions

The findings from this study contribute to the expanding evidence on the psychological effects of the COVID-19 pandemic on parents, especially fathers, highlighting its significant influence on paternal mental health and its repercussions in father–child relationships. This underscores the urgency of targeted interventions and support systems to respond to the mental health needs of parents during such unparalleled times, emphasizing the preservation of healthy parent–child interactions and bolstering family dynamics. Future research is imperative to explore the underlying mechanisms by which pandemic-induced stressors affect parental mental health and parenting practices, moving beyond merely identifying depressive symptoms to encompass a wider spectrum of psychological impacts, including anxiety and PTSD, that could influence parenting behaviors. Longitudinal studies are essential to monitor the enduring impacts of the pandemic on family structures and child development over time. Investigating the efficacy of mental health support interventions during crises, particularly for fathers who have received less attention in this area, becomes critical. By integrating diverse cultural perspectives and acknowledging the influence of maternal mental health on family interactions, we can enhance our comprehension and offer a comprehensive perspective on family well-being in the face of unprecedented challenges. The pandemic has underscored the pivotal importance of mental health within the family unit, highlighting the necessity for resources and strategies to assist parents in managing these difficulties.

## Figures and Tables

**Table 1:** Pearson correlation coefficients between the starting theoretical model variables.

	1.	2.	3.	4.
1. COVID-19 Risk Index	1			
2. CPDI	0.05	1		
3. DEPRESSIVE symptoms	0.06	0.41 **	1	
4. SVIA	0.06	0.43 **	0.03	1

Note. CPDI = COVID-19 Peritraumatic Distress Index; SCL-90/R = Symptom Checklist; SVIA = Feeding Scale; ** *p* < 0.01.

**Table 2:** Results of hierarchical multiple regression analyses predicting fathers’ depressive symptoms and quality of feeding interactions.

		Adjusted Coefficients		
		*B*	*t*	*p*
Predictors				
	COVID-19 Risk Index	0.16	3.05	0.002 **
	CPDI	0.09	1.72	0.008
	DEPRESSIVE symptoms	0.17	3.24	0.002 **
	SVIA	0.16	2.98	0.002 **
	R^2^			0.13
	R^2^ change			0.12

Note. CPDI = COVID-19 Peritraumatic Distress Index; SCL-90/R = Symptom Checklist; SVIA = Feeding Scale. ** *p* < 0.01.

## Data Availability

Data will be shared following reasonable request to the authors.
